# Tirzepatide as a Potential Disease-Modifying Therapy in Lipedema: A Narrative Review on Bridging Metabolism, Inflammation, and Fibrosis

**DOI:** 10.3390/ijms262110741

**Published:** 2025-11-05

**Authors:** Diogo Pinto da Costa Viana, Adriana Luckow Invitti, Eduardo Schor

**Affiliations:** 1 Department of Gynecology, Escola Paulista de Medicina, Federal University of Sao Paulo (EPM-UNIFESP), Sao Paulo 04024-002, Brazil; 2 Brazilian Society for Research and Teaching in Medicine (SOBRAPEM), Sao Paulo 01318-901, Brazil; 3 Brazilian Society of Obesity Medicine (SBEMO), Florianópolis 88070-800, Brazil

**Keywords:** lipedema, tirzepatide, Glucagon-Like Peptide-1 recptor agonist, adipose tissue dysfunction, inflammation, mitochondrial dysfunction

## Abstract

Lipedema is a chronic, progressive adipose tissue disorder that affects up to 10% of women and is characterized by disproportionate lower-limb fat accumulation, pain, edema, and resistance to conventional weight-loss approaches. Its pathophysiology involves a complex interplay of adipocyte hypertrophy, chronic inflammation, extracellular matrix fibrosis, mitochondrial dysfunction, and sex steroid imbalance, highlighting the need for disease-modifying therapies. This narrative review synthesizes mechanistic, translational, and clinical evidence linking metabolic, inflammatory, and fibrotic pathways to lipedema and tirzepatide’s potential therapeutic relevance. Tirzepatide, a dual GLP-1 (Glucagon-Like Peptide-1)/GIP (Glucose-Dependent Insulinotropic Polypeptide) receptor agonist, has demonstrated unprecedented efficacy in obesity and diabetes, alongside pleiotropic actions on inflammation, fibrosis, and adipose remodeling. Mechanistic studies reveal favorable effects on macrophage polarization, cytokine signaling, extracellular matrix turnover, and thermogenesis, suggesting potential relevance to lipedema biology. Translational evidence from related fibro-inflammatory conditions such as steatohepatitis and heart failure further supports its antifibrotic and immunomodulatory plausibility. Although direct clinical evidence in lipedema is lacking, the convergence of mechanistic pathways provides a strong rationale to investigate tirzepatide as a disease-modifying candidate. If future clinical studies confirm these mechanisms, tirzepatide could represent a novel metabolic–hormonal therapy capable of modifying the natural course of lipedema.

## 1. Introduction

Lipedema is a chronic, progressive, and underdiagnosed disorder of adipose tissue that affects almost exclusively women and may reach up to 10% of the female population worldwide [1,2,3,4,5]. It is characterized by symmetrical and disproportionate subcutaneous fat deposition in the limbs, often sparing the hands and feet, and is typically associated with pain, edema, and easy bruising [6,7,8]. Despite its high prevalence and considerable impact on functionality, emotional health, and social participation, lipedema is frequently misdiagnosed as obesity, lymphedema, or chronic venous insufficiency, which contributes to diagnostic delays and inappropriate therapeutic approaches [9,10].

The historical trajectory of lipedema research reflects the difficulty of distinguishing it from other adipose and vascular disorders. The early clinical descriptions by Allen and Hines (1940) [1] and Wold et al. (1951) [2] already emphasized its predominance in women, its hormonal association, and its resistance to caloric restriction, but for decades the condition remained poorly recognized outside specialized centers. Only in the last two decades has lipedema received renewed attention as a distinct adipose tissue disease, with better-defined diagnostic criteria and its inclusion in international classifications. This renewed interest has been driven by the recognition of its disabling impact on quality of life and its distinction from both primary obesity and lymphedema.

From a diagnostic perspective, lipedema requires careful differentiation from other conditions that produce limb enlargement. Obesity, for instance, produces generalized adipose accumulation but lacks the disproportion and pain typical of lipedema. Lymphedema is characterized by fluid retention and positive Stemmer’s sign, features absent in lipedema. Chronic venous insufficiency, although associated with edema and skin changes, does not reproduce the painful, fibrotic, and bruising-prone fat deposition observed in lipedema. This diagnostic overlap contributes to the frequent underrecognition of lipedema in clinical practice, which in turn delays adequate treatment and exposes patients to stigmatizing labels of being “obese” or “non-compliant” with dietary interventions.

The impact of lipedema on quality of life is profound. Studies document high levels of pain, functional impairment, mobility restrictions, and psychological distress, including depression and anxiety, in affected women [10]. Social stigma exacerbates this burden, as patients often encounter skepticism regarding the organic nature of their symptoms. The combination of physical disability, psychosocial stress, and economic cost, given the need for compression therapy, repeated consultations, and, in some cases, surgical procedures, places lipedema among the most neglected yet burdensome chronic conditions in women’s health.

The clinical course of the disease is strongly related to phases of hormonal transition such as puberty, pregnancy, and menopause, suggesting an important endocrine basis [11]. Recent studies point to alterations in estrogen signaling, including predominance of Estrogen Receptor beta (ERβ) over Estrogen Receptor alpha (ERα), as well as intracrine estradiol production mediated by aromatase and 17β-Hydroxysteroid Dehydrogenase (17β-HSD), which play a central role in lipedematous adipose tissue dysfunction [12,13,14]. These mechanisms create a chronic inflammatory microenvironment infiltrated by M1 macrophages, associated with intense fibrosis and microvascular alterations, thereby generating a cycle of disease perpetuation resistant to conventional therapies [7,15,16].

A hallmark of lipedema is the resistance of affected adipose tissue to traditional fat-reducing strategies. Caloric restriction and physical activity, which typically reduce fat mass in obesity, are largely ineffective in lipedema [17,18]. Even bariatric surgery, a powerful treatment for obesity, does not change the disproportionate fat distribution characteristic of the disease [19]. Similarly, anti-obesity pharmacological agents have not produced consistent results in this setting, revealing the need for new therapeutic approaches [19].

In recent years, the growing understanding of immunometabolic and hormonal mechanisms in lipedema has opened avenues for more targeted pharmacological strategies. Incretin-based therapies have emerged as promising candidates. A pilot Italian study evaluated the GLP-1 agonist exenatide in women with lipedema and insulin resistance, suggesting both metabolic and clinical benefits [20,21]. Although preliminary, these findings support the concept that incretin signaling may address key pathways of lipedema pathophysiology. Among available agents, tirzepatide, a dual GLP-1 and GIP agonist, offers broader and more potent actions.

Tirzepatide has demonstrated superior effects on weight reduction and glycemic control compared with single GLP-1 agonists [22,23]. Beyond weight loss, preclinical and clinical studies have documented anti-inflammatory, antifibrotic, and adipose remodeling properties, including increased Uncoupling Protein-1 (UCP1) mediated thermogenesis through activation of beige adipocytes and restoration of mitochondrial efficiency., which directly overlap with lipedema’s pathogenic mechanisms [21,24,25,26,27,28]. Given its dual GLP-1/GIP agonism, tirzepatide emerges as the most plausible pharmacological candidate capable of interrupting the pathogenic cycle of lipedema and potentially modifying the natural history of the disease.

This review adopts a translational, hypothesis-driven perspective, integrating mechanistic and clinical evidence on lipedema biology and tirzepatide plausibility. The synthesis is narrative in nature, without systematic or quantitative analysis, and was developed through a comprehensive literature search of PubMed/MEDLINE, Scopus, and Web of Science databases for English-language articles published between 2010 and 2025 using combinations of the following keywords: “lipedema,” “tirzepatide,” “GLP-1,” “GIP,” “fibrosis,” and “adipose tissue”. Reference lists of relevant papers were also reviewed to identify additional sources. Both preclinical and clinical studies exploring metabolic, inflammatory, and fibrotic mechanisms were included, while case reports, non-English publications, and unrelated experimental studies were excluded.

## 2. Pathophysiological Mechanisms of Lipedema

### 2.1. Adipose Tissue Dysfunction and Inflammation

Lipedema is increasingly recognized as a distinct adipose tissue disorder whose pathological features set it apart from both obesity and normal adiposity. Histological and molecular studies consistently demonstrate hypertrophy of adipocytes, excessive extracellular matrix deposition, and dense interstitial fibrosis, which together impart stiffness to the tissue and pain upon palpation [7,8]. These morphological changes are not isolated findings; rather, they are part of a broader disturbance in the structural and immune microenvironment of subcutaneous adipose tissue. Increased microvascular permeability and the presence of lymphatic microangiopathies have been documented, favoring interstitial edema, local hypoxia, and compromised nutrient exchange [15]. This constellation of features creates a pathological substrate for disease chronicity.

Within this altered microenvironment, inflammatory pathways are persistently activated. Pro-inflammatory M1 macrophages accumulate in the adipose depots of affected limbs, secreting cytokines such as Tumor Necrosis Factor-alpha (TNF-α), Interleukin-6 (IL-6), and Monocyte Chemoattractant Protein-1 (MCP-1). These cytokines sustain low-grade chronic inflammation, recruit additional immune cells, and perpetuate vascular dysfunction [6,16]. Parallel fibroblast activation leads to ongoing extracellular matrix remodeling, excessive collagen deposition, and expression of fibrotic markers such as YKL-40, as demonstrated in recent analyses of lipedematous tissue [25,26]. Together, inflammation and fibrosis establish a self-reinforcing cycle that explains both the pain and the resistance to conventional weight-loss approaches. Importantly, these mechanisms—particularly macrophage-driven inflammation and fibrotic remodeling—overlap with immunometabolic targets that have been shown to respond to incretin-based interventions, including tirzepatide, thereby reinforcing the translational rationale for its use.

### 2.2. Hormonal Dysregulation and Intracrine Estradiol Production

The clinical expression of lipedema is closely tied to hormonal transitions, appearing or worsening during puberty, pregnancy, and menopause. This correlation underscores the central role of sex steroid signaling in the disease pathophysiology [11]. Several investigations highlight a consistent imbalance between estrogen receptor subtypes, with predominance of ERβ over ERα in affected adipose depots. Such receptor imbalance is associated with adipocyte hypertrophy, pro-inflammatory signaling, and fibrotic remodeling, further exacerbating disease burden [14,21].

Beyond receptor expression, intracrine mechanisms magnify local estrogen exposure. Increased aromatase activity enhances androgen-to-estradiol conversion, while reduced 17β-HSD2 expression impairs estradiol inactivation, leading to persistent hormonal activity within adipose tissue [12]. These alterations generate local estradiol excess not reflected in circulating hormone levels, explaining why many patients show normal laboratory profiles despite profound tissue dysfunction. Intracrine estradiol enrichment, in combination with ERβ predominance, creates a biologically active microenvironment that promotes adipocyte proliferation, abnormal angiogenesis, and further fibrotic remodeling. The recognition that lipedema progression is driven by these local hormonal imbalances reinforces the plausibility of therapies that indirectly modulate these pathways, such as incretin agonists, by improving metabolic homeostasis and reducing inflammation.

### 2.3. Progesterone Resistance

Resistance to progesterone signaling represents another critical hormonal mechanism in lipedema. Altered expression and function of progesterone receptors diminish the hormone’s normal anti-inflammatory and antiproliferative actions, leading to unchecked inflammation and fibrosis [12]. This phenomenon also explains the overlap between lipedema and gynecological disorders such as endometriosis and adenomyosis, which share similar progesterone resistance. In lipedema, progesterone resistance translates into loss of protective modulation against estradiol-driven pathways, intensifying adipocyte hypertrophy and fibrotic progression. Recognizing this hormonal resistance underscores the need for therapies capable of counteracting inflammation and fibrosis. Tirzepatide, by modulating immune polarization and reducing cytokine burden, could attenuate downstream consequences of this resistance, offering a rationale for testing in patient subsets where progesterone dysfunction is most pronounced.

### 2.4. Metabolic and Mitochondrial Dysfunction

From a metabolic perspective, lipedema tissue demonstrates rigidity and an impaired capacity for lipid mobilization. Even under caloric restriction or increased physical activity, the affected depots fail to reduce in volume. Mitochondrial studies show reduced oxidative capacity and downregulation of thermogenic proteins such as UCP1, which play critical roles in energy expenditure [6]. This metabolic inflexibility explains why women with lipedema often fail to achieve limb volume reduction despite global weight loss. Insulin resistance and dysregulated lipid metabolism further complicate disease progression, especially in perimenopausal and postmenopausal women, in whom declining estradiol is coupled with visceral fat gain, systemic inflammation, and worsening metabolic syndrome [29]. Tirzepatide directly addresses these mechanisms by enhancing insulin sensitivity, upregulating UCP1, activating beige adipocytes, and restoring adipose tissue flexibility [24]. These actions align with key pathogenic nodes of lipedema biology, strengthening the mechanistic rationale for its therapeutic exploration.

### 2.5. Resistance to Conventional Therapies

A hallmark of lipedema is its refractoriness to conventional obesity therapies. Hypocaloric diets typically reduce visceral and truncal fat but have little impact on disproportionate lower-limb adiposity [17]. Bariatric surgery, effective for obesity, results in global weight reduction and metabolic improvement, yet lipedema fat distribution remains unchanged, with minimal impact on symptoms or quality of life [18]. Pharmacological treatments approved for obesity, including orlistat, bupropion–naltrexone, and GLP-1 receptor agonists such as semaglutide, have also shown limited efficacy in modifying lipedema depots [19]. A small pilot trial with the GLP-1 agonist exenatide long-acting release formulation in women with lipedema and insulin resistance reported modest clinical and metabolic improvements, but its small sample size and observational design limit generalizability [21]. Liposuction remains the only intervention consistently associated with measurable limb volume reduction, but it is invasive, carries surgical risks, and does not address underlying pathophysiology, allowing disease progression to continue [19,30].

The refractoriness to established therapies highlights lipedema as more than an energy imbalance disorder, positioning it instead as a condition rooted in metabolic, immunological, and hormonal dysregulation. In this context, tirzepatide, through dual GLP-1/GIP agonism and pleiotropic effects, emerges as a rational candidate capable of addressing the intertwined mechanisms that sustain disease progression.

### 2.6. Integrative Synthesis

Collectively, these findings reinforce that lipedema is not simply a disorder of excessive adiposity but a complex immunometabolic disease with unique biological hallmarks. Chronic inflammation, fibrosis, mitochondrial dysfunction, and hormonal imbalance drive its clinical course and explain its resistance to conventional approaches. Table 1 synthesizes the main mechanisms of lipedema and their overlap with tirzepatide’s actions, highlighting mechanistic evidence, translational plausibility, and proposed biomarkers and clinical endpoints for future clinical trials.

## 3. Tirzepatide as a Therapeutic Prospect in Lipedema

### 3.1. Mechanistic Rationale for Disease Modification

The synthesis of current evidence suggests that tirzepatide is not merely another agent for weight reduction but rather a therapy with the potential to influence the fundamental biology of lipedema. Its dual GLP-1 and GIP receptor agonism provides superior efficacy in metabolic endpoints compared with single agonists [22,23]. These systemic benefits address insulin resistance, visceral adiposity, and chronic inflammation, all of which amplify adipose tissue dysfunction and symptom burden in lipedema.

The metabolic impact of tirzepatide is particularly noteworthy. In phase III clinical trials, patients receiving tirzepatide achieved significantly greater reductions in both body weight and glycated hemoglobin compared with GLP-1 receptor agonists alone, underscoring its unique pharmacological profile. In SURMOUNT-1, participants with obesity experienced mean weight loss exceeding 20% after 72 weeks, an unprecedented result for an incretin-based therapy [23]. In the SURPASS-2 trial, patients with type 2 diabetes demonstrated not only substantial weight reduction but also a mean decrease of 2.3% in glycated hemoglobin, highlighting significant improvements in both glycemic control and adiposity-related endpoints [22]. These outcomes are highly relevant for lipedema, where systemic insulin resistance and metabolic inflexibility perpetuate inflammation and pain, and often coexist with comorbid conditions such as polycystic ovary syndrome.

Equally important is the pleiotropic profile of tirzepatide. Beyond appetite regulation and glycemic control, preclinical and clinical findings suggest that tirzepatide exerts actions that directly overlap with the pathological hallmarks of lipedema, including low-grade inflammation, fibrosis, and impaired adipose remodeling. The ability of tirzepatide to induce beige adipocyte activation and increase UCP1-mediated thermogenesis [24] aligns with the metabolic rigidity described in lipedematous tissue [6]. Furthermore, evidence from translational studies in metabolic dysfunction-associated steatohepatitis and cardiovascular disease demonstrates reductions in inflammatory and fibrotic signaling pathways, processes that are central to the progression of lipedema [28,32].

Taken together, the dual metabolic and immunomodulatory actions of tirzepatide reinforce its plausibility as a disease-modifying candidate for lipedema. While current data are extrapolated from obesity and diabetes populations, the overlap between mechanisms targeted by tirzepatide and those driving lipedema pathophysiology provides a strong translational rationale for future clinical testing. Table 1 provides a structured overview of the main pathophysiological mechanisms of lipedema, the corresponding evidence of tirzepatide action, and potential biomarkers or clinical endpoints for future evaluation. Insulin sensitivity-based lipedema originates from hormonal dysregulation, particularly the predominance of ERβ over ERα, intracrine estradiol excess, and progesterone resistance. The downstream consequences of these alterations produce a distinct adipose tissue phenotype characterized by chronic inflammation, macrophage polarization imbalance, extracellular matrix fibrosis, and mitochondrial dysfunction [12,13,14,25,26,27,28]. These secondary immunometabolic abnormalities constitute an independent therapeutic target. Tirzepatide, through dual GLP-1 and GIP receptor agonism, has demonstrated the ability to attenuate pro-inflammatory cytokine signaling, promote M2 macrophage polarization, enhance mitochondrial oxidative capacity, and partially reverse fibrosis, as shown in metabolic-associated steatohepatitis (MASH) and heart failure models [24,27,28,32]. By improving adipose tissue function rather than directly modifying sex-steroid receptors, tirzepatide could counteract the pathogenic consequences of hormonal imbalance that sustain lipedema progression. However, these antifibrotic effects are likely limited to early or moderate stages, where tissue remodeling remains partially reversible.

### 3.2. Anti-Inflammatory and Immunomodulatory Effects

Lipedema tissue is characterized by macrophage M1 predominance, local secretion of TNF-α, IL-6, and MCP-1, and an immune microenvironment that sustains chronic low-grade inflammation [6,16]. This pro-inflammatory milieu is not a passive finding but rather a driving force behind disease progression, contributing to both adipose tissue rigidity and pain sensitization. Chronic macrophage activation perpetuates extracellular matrix remodeling, enhances vascular leakage, and fuels fibroblast activity, thereby linking inflammation directly to the fibrotic burden that characterizes advanced stages of lipedema.

Experimental data show that incretin signaling can shift macrophage polarization toward an anti-inflammatory M2 phenotype, reducing cytokine release and restoring tissue homeostasis [24,31]. This immunomodulatory action extends beyond macrophages, as incretin pathways have also been implicated in reducing recruitment of monocytes and T lymphocytes to inflamed adipose depots. By rebalancing the immune microenvironment, tirzepatide could theoretically alleviate the chronic pain and stiffness described by patients, symptoms closely tied to inflammatory infiltration of subcutaneous fat.

The potential relevance of these effects becomes particularly clear in women with concomitant polycystic ovary syndrome, a phenotype characterized by insulin resistance and systemic inflammation that often coexists with lipedema. In such cases, the inflammatory and metabolic axes converge, amplifying disease severity. By simultaneously improving insulin sensitivity and dampening inflammatory cytokine release, tirzepatide may exert dual benefits, reducing systemic drivers while also acting on local adipose depots [21].

In this sense, the anti-inflammatory potential of tirzepatide should not be viewed solely as a secondary consequence of weight loss. Instead, it represents a direct pharmacological action with potential to modify one of the central pathogenic cycles of lipedema: the persistence of an immune-dysregulated, fibrotic, and painful adipose microenvironment. Although clinical validation in lipedema cohorts remains absent, the convergence of mechanistic evidence underscores tirzepatide as a rational candidate to test in inflammatory-driven adipose diseases.

Recent transcriptomic and immunophenotypic analyses confirmed a predominance of M2-like macrophages in lipedema adipose tissue [34,35]. However, these M2 cells display a metabolically activated, profibrotic phenotype characterized by increased expression of extracellular matrix–remodeling and angiogenic genes rather than the anti-inflammatory properties observed in healthy adipose tissue. Thus, therapeutic strategies aiming to restore balanced macrophage function by reprogramming M2-like cells toward a resolutive, antifibrotic state, remain mechanistically sound. Dual GIP/GLP-1 agonists such as tirzepatide have been shown to induce this shift in macrophage phenotype, suppressing profibrotic signaling and enhancing metabolic homeostasis in preclinical models [21,27].

### 3.3. Antifibrotic and Extracellular Matrix Remodeling

Dense interstitial fibrosis is one of the histological hallmarks of lipedema, consistently observed in both early and advanced stages of the disease [7,25,26]. Fibrosis not only stiffens the adipose tissue and contributes to chronic pain, but it also creates a hostile environment that limits oxygen diffusion, impairs vascular integrity, and reduces the effectiveness of conventional therapeutic approaches. The excessive extracellular matrix (ECM) deposition also traps inflammatory cells within adipose depots, thereby perpetuating a cycle of chronic inflammation and tissue remodeling that accelerates disease progression and functional impairment.

Evidence from other fibrotic and metabolic diseases has demonstrated that tirzepatide can exert antifibrotic effects. In patients with metabolic-associated steatohepatitis (MASH), tirzepatide therapy was associated with significant reductions in inflammatory markers and fibrosis scores, providing proof that incretin pathways can attenuate pathological ECM remodeling in human tissues [27]. Likewise, in experimental models of heart failure with preserved ejection fraction (HFpEF), tirzepatide improved cardiac structure and function while reducing interstitial fibrosis, highlighting its capacity to influence fibroblast activity and matrix deposition in non-adipose tissues [28].

The plausibility of these effects in lipedema rests on the shared biology of fibrosis across different organs. Fibroblast activation, excessive collagen synthesis, and matrix cross-linking are not restricted to the liver or heart but are also prominent in subcutaneous adipose depots affected by lipedema. By reducing profibrotic signaling and dampening chronic ECM accumulation, tirzepatide may be able to restore at least partial tissue flexibility, improve microvascular exchange, and mitigate functional limitations in affected limbs.

Although direct evidence in lipedema is lacking, the convergence of mechanistic and clinical findings in other fibrotic conditions supports the rationale for testing tirzepatide as a modulator of tissue remodeling in this disease. At the same time, extrapolation must be undertaken with caution, since the fibrotic response in lipedema involves a unique interplay of hormonal imbalance, intracrine estradiol activity, and immune dysregulation. Nonetheless, the overlap of pathways affected by tirzepatide with those central to lipedema pathophysiology strengthens the case for targeted clinical trials designed to determine whether antifibrotic actions can translate into tangible clinical improvements.

### 3.4. Adipose Remodeling and Mitochondrial Flexibility

Mitochondrial dysfunction and impaired thermogenesis are central hallmarks of lipedema adipose tissue, contributing to its rigidity and inability to mobilize stored lipids effectively [6]. Affected depots demonstrate reduced oxidative phosphorylation capacity and downregulation of key thermogenic proteins such as UCP1, which are normally critical for adaptive energy expenditure. This lack of metabolic flexibility explains why patients with lipedema often fail to achieve proportional limb fat reduction despite global weight loss induced by diet, exercise, or even bariatric surgery [17,18]. As a result, the disease is perpetuated not only by caloric imbalance but also by intrinsic cellular defects in energy handling.

Preclinical studies have provided compelling evidence that tirzepatide may counteract these deficits. By simultaneously activating GLP-1 and GIP receptors, tirzepatide enhances mitochondrial function, increases UCP1 expression, and promotes the emergence of beige adipocytes from white adipose depots [24]. These beige adipocytes are metabolically more active and capable of dissipating energy through thermogenesis, thereby restoring a degree of metabolic plasticity that is absent in lipedema tissue. The induction of thermogenesis and remodeling of adipose depots could reduce the pathologic energy trapping that characterizes lipedema, facilitating not only weight loss but also changes in body composition more relevant to the disease phenotype.

This mitochondrial remodeling is particularly relevant for women with lipedema in peri- and postmenopausal stages, where reduced estradiol levels exacerbate visceral fat accumulation, systemic inflammation, and insulin resistance [13]. In such contexts, tirzepatide’s ability to improve insulin sensitivity and reactivate mitochondrial pathways offers a mechanistic solution to the metabolic rigidity that conventional therapies cannot overcome. Restoring mitochondrial efficiency may also alleviate tissue hypoxia, reduce reactive oxygen species accumulation, and secondarily decrease inflammatory signaling, thereby linking metabolic and immunological improvements.

While clinical confirmation in lipedema is still lacking, the alignment of preclinical findings with central defects in lipedema adipose biology provides a strong rationale for exploration. If tirzepatide can restore thermogenic capacity and adipose flexibility in affected depots, it may address one of the most entrenched barriers to effective management of the disease. The potential to shift dysfunctional white adipose tissue toward a more metabolically adaptable phenotype underscores tirzepatide’s plausibility as a therapy that acts not only on systemic energy balance but also directly on the cellular machinery of lipedema tissue.

Although physiological GIP signaling can favor lipid deposition in subcutaneous depots under conditions of normal insulin sensitivity [36], tirzepatide’s dual agonism fundamentally redefines this response. By co-activating GLP-1 receptors, tirzepatide shifts GIP signaling from anabolic toward catabolic and anti-inflammatory pathways, enhancing adipocyte insulin sensitivity, mitochondrial oxidation, and tissue remodeling [27,37]. This context-dependent reprogramming explains why tirzepatide, unlike isolated GIP exposure, produces net reductions in subcutaneous and visceral fat even in insulin-sensitive phenotypes such as early-stage lipedema.

### 3.5. Translational Evidence from Related Conditions

Large-scale clinical trials have consistently demonstrated the systemic efficacy of tirzepatide, offering indirect but compelling evidence that its mechanisms may be relevant to lipedema. In the SURMOUNT-1 trial, patients with obesity achieved a mean body weight reduction of 20.9% after 72 weeks at the 15 mg dose, a magnitude of weight loss unprecedented among currently available pharmacological options [23]. Importantly, this degree of reduction not only reflects decreased caloric intake but also suggests substantial improvements in metabolic regulation and adipose tissue biology. Similarly, in the SURPASS-2 trial involving patients with type 2 diabetes, tirzepatide induced HbA1c reductions of up to −2.3% and an average weight loss of 11.2 kg, underscoring its ability to improve glycemic control and counteract systemic insulin resistance [22]. Both obesity and type 2 diabetes share with lipedema a background of chronic inflammation, adipocyte dysfunction, and metabolic inflexibility, making these results particularly relevant.

Beyond weight and glycemic endpoints, tirzepatide has shown benefits in conditions characterized by inflammation and fibrosis, which are central features of lipedema. In MASH, treatment with tirzepatide significantly reduced hepatic inflammation and fibrosis, demonstrating its capacity to modulate tissue remodeling in a chronic fibro-inflammatory disease [32]. Similarly, in experimental models of HFpEF, tirzepatide improved cardiac remodeling, reduced interstitial fibrosis, and enhanced overall cardiac function [28]. These findings illustrate that tirzepatide exerts antifibrotic and anti-inflammatory actions across different organ systems, reinforcing the hypothesis that its mechanisms may be transferable to lipedema, where fibrosis and chronic low-grade inflammation are key drivers of progression.

Taken together, these data suggest that tirzepatide is not confined to metabolic regulation alone but also exerts broader biological effects that intersect with the pathogenic nodes of lipedema. The consistency of benefits across diverse conditions, ranging from obesity and type 2 diabetes to MASH and HFpEF, supports the idea that tirzepatide acts on shared molecular pathways, including mitochondrial flexibility, immune modulation, and extracellular matrix remodeling. Although direct clinical evidence in lipedema remains absent, the translational plausibility is strong: if tirzepatide can reverse fibro-inflammatory remodeling in hepatic and cardiac tissue, it may similarly impact the pathological fibrosis and immune imbalance of lipedema depots.

For this reason, these findings provide a solid foundation to prioritize well-designed lipedema-specific trials. The extrapolation from related conditions should not be considered confirmatory but rather as a strong rationale that tirzepatide targets fundamental mechanisms also present in lipedema. A chronological summary of the main clinical and preclinical studies supporting these mechanisms is provided in Table 2. This cross-disease evidence strengthens the argument that tirzepatide could represent the first pharmacological approach capable of addressing the multifactorial biology of lipedema.

### 3.6. Gynecological Interface and Dual Benefits

The overlap between lipedema and estrogen-dependent gynecological disorders, including polycystic ovary syndrome (PCOS), endometriosis, adenomyosis, and uterine fibroids, is increasingly well documented and represents an important clinical intersection [12,20]. These conditions not only coexist epidemiologically but also share overlapping mechanisms of pathophysiology, including progesterone resistance, intracrine estradiol excess, and predominance of ERβ over ERα in target tissues [11,14]. Such alterations contribute to abnormal tissue growth, inflammation, and fibrosis, processes that parallel those observed in lipedema depots. This convergence suggests that gynecological comorbidities should not be considered separate entities in affected women but rather as phenotypic extensions of shared hormonal and metabolic disturbances.

Recent reviews have highlighted the potential role of incretin-based therapies in hormone-dependent gynecological diseases, given their ability to modulate inflammation, metabolism, and tissue remodeling [33]. The biological plausibility of applying tirzepatide in this context arises from its dual GLP-1/GIP agonism, which simultaneously improves systemic insulin sensitivity and attenuates chronic inflammation. In women with PCOS, for example, hyperinsulinemia and insulin resistance drive hyperandrogenism, adipose dysfunction, and reproductive disturbances. By reducing insulin resistance and systemic inflammation, tirzepatide may indirectly ameliorate these pathways, potentially exerting dual benefits in women with lipedema complicated by PCOS.

Similarly, in endometriosis and adenomyosis, chronic inflammation and progesterone resistance play pivotal roles in disease persistence. The ability of tirzepatide to reduce pro-inflammatory cytokines, alter macrophage polarization, and modulate fibrotic remodeling [24,31] suggests it could influence the microenvironment of endometriotic lesions and potentially mitigate symptom severity. Although direct clinical trials in gynecological diseases are lacking, the shared pathophysiological basis creates a compelling rationale to test incretin-based strategies in these conditions.

In the menopausal transition, the overlap becomes even more pronounced. Women with lipedema frequently report worsening symptoms after menopause, coinciding with estradiol decline, visceral fat gain, and increased systemic inflammation [29]. These processes converge with metabolic syndrome, cardiovascular risk, and progression of gynecological comorbidities. Tirzepatide, by addressing insulin resistance, visceral adiposity, and chronic inflammation, could provide therapeutic synergy, offering benefits that extend beyond lipedema to the broader spectrum of postmenopausal health challenges.

Altogether, the gynecological interface strengthens the translational rationale for tirzepatide in lipedema. Rather than being viewed solely as a metabolic drug, tirzepatide could function as a bridge between endocrinology and gynecology, targeting shared pathways across adipose, reproductive, and metabolic tissues. Such a perspective justifies the inclusion of gynecological stratification in future lipedema trials, enabling the identification of subgroups such as women with PCOS, endometriosis, or menopausal status, in which tirzepatide may yield dual or even synergistic benefits. This integrative framework not only enhances the plausibility of tirzepatide as a candidate therapy but also positions it at the crossroads of two major fields in women’s health.

### 3.7. Clinical and Research Implications

The persistent refractoriness of lipedema to standard therapies underscores the urgency of exploring innovative pharmacological approaches. Current strategies, dietary restriction, bariatric surgery, pharmacological agents for obesity, and even liposuction, fail to modify the underlying biology of the disease, leaving a critical therapeutic gap [17,18,19]. Tirzepatide emerges as a strong candidate to fill this gap due to its integrated effects on systemic metabolism, inflammation, fibrosis, and adipose remodeling, which align with the principal mechanisms identified in lipedema pathology.

From a clinical perspective, several implications arise. First, randomized controlled trials (RCTs) will be essential to evaluate tirzepatide’s impact specifically in lipedema, since current evidence is largely extrapolated from obesity, type 2 diabetes, MASH, and HFpEF [22,23,27,28]. Future RCTs should move beyond weight reduction as the primary endpoint and incorporate disease-relevant outcomes such as lower-limb volume assessed by magnetic resonance imaging, quantification of fibrotic tissue through histological or imaging biomarkers, and objective evaluation of inflammation using circulating cytokines or tissue-based markers. Patient-reported outcomes, including pain, functional mobility, and quality of life, must be integrated to capture the multidimensional impact of lipedema.

Second, stratification of participants is critical. Lipedema frequently coexists with gynecological comorbidities such as PCOS, endometriosis, and adenomyosis, which may influence both disease expression and response to therapy [12,20]. Similarly, menopausal status modifies metabolic and inflammatory profiles, potentially altering treatment efficacy [29]. Trials designed with subgroup analyses could help determine whether tirzepatide’s benefits are uniform across the spectrum of lipedema phenotypes or whether certain groups—such as premenopausal women with PCOS or postmenopausal women with metabolic syndrome derive greater advantages. This stratified approach aligns with precision medicine principles and could refine therapeutic recommendations.

Third, long-term safety and durability of effect require careful evaluation. While data from obesity and diabetes trials demonstrate favorable cardiometabolic safety profiles, lipedema patients may present unique vulnerabilities, including connective tissue fragility and chronic pain syndromes. Registries and longitudinal extension studies could provide insights into sustained efficacy, cardiovascular safety, preservation of lean mass, and potential effects on gynecological outcomes.

Finally, combination strategies represent an important frontier. Tirzepatide may act synergistically with existing interventions, including compression therapy, manual lymphatic drainage, physiotherapy, and even hormonal modulators such as gestrinone or drospirenone, which target overlapping mechanisms of adipose and gynecological pathology. The possibility of integrating pharmacological and mechanical strategies opens the way to multimodal management protocols that could more effectively control both symptoms and progression of lipedema.

**Figure 1:**
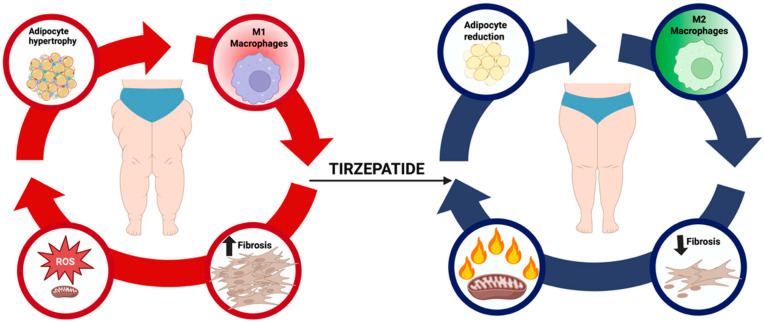
Conceptual model of tirzepatide in lipedema. The pathogenic cycle of lipedema links adipocyte hypertrophy, macrophage-driven inflammation, extracellular matrix (ECM) fibrosis, and excessive generation of Reactive Oxygen Species (ROS), leading to mitochondrial dysfunction, pain, edema, and therapeutic resistance. Tirzepatide, a dual co-agonist of the Glucose-Dependent Insulinotropic Polypeptide (GIP) and Glucagon-Like Peptide-1 (GLP-1) receptors, is hypothesized to attenuate adipocyte hypertrophy, shift macrophage polarization toward an anti-inflammatory phenotype, reduce profibrotic signaling and ECM accumulation, and restore thermogenesis through Uncoupling Protein-1 (UCP1) upregulation. By targeting these interrelated nodes, tirzepatide may plausibly disrupt disease progression and provide a disease-modifying benefit.

In sum, tirzepatide offers a unique translational opportunity to reshape the therapeutic landscape of lipedema. However, rigorous clinical validation is indispensable before widespread clinical adoption. The challenge for the next phase of research will be to design trials that are mechanistically informed, patient-centered, and sufficiently powered to provide definitive answers. By addressing these priorities, the field can determine whether tirzepatide truly represents the first pharmacological candidate capable of modifying the natural history of lipedema and improving outcomes for the millions of women affected worldwide. Figure 1 illustrates the hypothesized disease-modifying role of tirzepatide in lipedema, highlighting its effects on adipocyte hypertrophy, macrophage polarization, and fibrosis.

By integrating these findings, it becomes clear that lipedema is sustained by a network of interrelated mechanisms, chronic inflammation, fibrosis, mitochondrial dysfunction, and hormonal imbalance, that reinforce one another and explain the disease’s resistance to conventional interventions. Tirzepatide, by addressing several of these nodes simultaneously through metabolic, immunological, and antifibrotic actions, emerges as a plausible candidate for disease modification. Although current evidence remains extrapolated from related conditions, this multidimensional overlap provides a coherent rationale for prioritizing dedicated clinical trials. Given that tirzepatide is not approved for the treatment of lipedema, any potential use should be considered off-label and restricted to research settings or compassionate use with full informed consent, ethics approval, and appropriate safety monitoring.

### 3.8. Safety, Tolerability, and Risk–Benefit Considerations

The safety profile of tirzepatide has been extensively characterized across large clinical programs involving obesity, type 2 diabetes, and metabolic-associated steatohepatitis (MASH). In pooled analyses from the SURPASS and SURMOUNT trials, the most common adverse events were gastrointestinal, including nausea (20–25%), diarrhea (15–20%), and vomiting (5–8%), typically mild to moderate and transient [22,23]. Discontinuation rates due to adverse effects were below 8%, comparable to or lower than those observed with GLP-1 receptor agonists. Hypoglycemia was rare and occurred primarily in combination with insulin or sulfonylureas.

Cardiometabolic safety outcomes have been consistently favorable. Tirzepatide reduced systolic blood pressure, improved lipid profiles, and showed no increase in major adverse cardiovascular events (MACE) compared with placebo or active comparators [22,23,28]. In the recent HFpEF trial, tirzepatide not only improved functional capacity but also reduced interstitial fibrosis and systemic inflammation without cardiovascular safety signals [28].

With respect to fibrosis-associated conditions, data from MASH models demonstrated attenuation of hepatic inflammation and fibrosis without evidence of hepatotoxicity [32]. No increase in pancreatitis or thyroid C-cell pathology has been reported in humans, and overall tolerability is considered excellent for long-term use.

From a translational perspective, these safety findings are particularly relevant to women with lipedema, who frequently present with comorbid metabolic syndrome, cardiovascular risk factors, and connective tissue fragility. The low incidence of serious adverse events and favorable cardiometabolic effects strengthens the case for clinical exploration in this population.

Overall, tirzepatide offers a robust risk–benefit profile characterized by high efficacy in metabolic endpoints and an acceptable safety margin, supporting its candidacy as a potential disease-modifying therapy in lipedema.

## 4. Conclusions

Lipedema remains a chronic, disabling, and underdiagnosed disorder that continues to challenge clinicians due to its resistance to conventional therapies and its frequent overlap with gynecological and metabolic comorbidities. Current strategies, ranging from dietary restriction to bariatric surgery and liposuction, have demonstrated limited and inconsistent efficacy, underscoring the urgent need for therapies that act on the underlying pathophysiological processes rather than on symptom management alone.

Tirzepatide, through its dual GLP-1/GIP agonism, has emerged as a particularly compelling candidate to explore in this context. Beyond its capacity to induce unprecedented weight reduction and glycemic improvement in large randomized trials, tirzepatide exhibits pleiotropic effects on inflammation, fibrosis, and adipose remodeling that align with the central mechanisms described in lipedema. Translational findings from metabolic-associated steatohepatitis and heart failure models provide further plausibility that pathways relevant to lipedema biology could be drug-responsive. Nonetheless, at this stage, these signals should be regarded as hypothesis-generating rather than definitive.

The absence of randomized controlled trials in lipedema represents the most critical limitation, and caution is warranted against premature clinical extrapolation. Well-designed studies are needed to determine whether tirzepatide can meaningfully alter the course of lipedema, ideally incorporating imaging-based assessment of limb volume, molecular biomarkers of inflammation and fibrosis, patient-reported pain and quality-of-life outcomes, and stratification by gynecological phenotypes and menopausal status. The identification of predictive biomarkers, such as ERβ predominance, intracrine estradiol activity, or macrophage polarization profiles, may help select subgroups most likely to benefit.

In addition to these unmet needs, several critical research gaps persist: the lack of validated histological biomarkers for diagnosis and monitoring, the absence of long-term metabolic and functional follow-up in affected cohorts, and the lack of standardized clinical endpoints specific to lipedema across trials. Addressing these gaps will be essential to ensure rigorous evaluation of any emerging therapy.

If future studies validate its effects in this indication, tirzepatide could represent the first pharmacological approach capable of modifying the natural history of lipedema, potentially shifting management from symptomatic interventions toward disease-targeted therapy. For this transformation to occur, collaborative multicenter efforts, harmonized outcome measures, and integration with ongoing research in gynecological endocrinology and metabolic disease will be essential.

In conclusion, tirzepatide emerges as a scientifically plausible and promising candidate for disease-targeted therapy in lipedema, warranting rigorous evaluation in well-designed clinical studies. Establishing whether its metabolic, antifibrotic, and immunomodulatory properties can translate into tangible clinical benefits for patients with lipedema remains a critical and timely research priority.

## Figures and Tables

**Table 1:** Summary of the main pathophysiological mechanisms involved in lipedema and corresponding evidence of tirzepatide action. Abbreviations: GIP, Glucose-Dependent Insulinotropic Polypeptide; GLP-1, Glucagon-Like Peptide-1; T2D, type 2 diabetes; MASH, metabolic dysfunction-associated steatohepatitis; HFpEF, heart failure with preserved ejection fraction; UCP1, Uncoupling Protein-1; PCOS, polycystic ovary syndrome.

Domain	Key Finding (Mechanism/Clinical)	Model or Population	Relevance to Lipedema Biology
Metabolic control [22,23]	Dual co-agonism of Glucose-Dependent Insulinotropic Polypeptide (GIP) and Glucagon-Like Peptide-1 (GLP-1) receptors produces greater weight loss and glycemic improvement compared to single GLP-1 receptor agonism.	Phase III clinical trials in obesity and type 2 diabetes (T2D)	Addresses systemic insulin resistance that amplifies adipose inflammation and pain burden in lipedema.
Inflammation (macrophages) [24,31]	Incretin signaling shifts macrophage polarization toward anti-inflammatory M2 phenotype and reduces pro-inflammatory cytokines, counteracting M1-dominantinflammation.	Experimental/mechanistic data	Counteracts M1-dominant adipose inflammation described in lipedema tissue.
Fibrosis/Extracellular matrix (ECM) remodeling [28,32]	Tirzepatide reduces fibrotic signaling and tissue fibrosis in disease models.	Clinical (metabolic dysfunction-associated steatohepatitis—MASH) and experimental cardiac/heart failure with preserved ejection fraction (HFpEF)models	Fibrosis is a core histologic hallmark in lipedema; antifibrotic action is central to disease modification.
Adipose remodeling/thermogenesis [24]	Upregulates Uncoupling Protein-1 (UCP1) and activates beige adipocytes, increasing thermogenesis and energy expenditure.	Preclinical adipose studies	Directly targets mitochondrial rigidity and impaired fat mobilization found in lipedema depots.
Microvascular/stromal milieu [24,25,26]	Incretin pathways attenuate inflammatory-fibrotic crosstalk in adipose-rich tissues.	Integrative/mechanistic evidence	Aligns with microangiopathy and matrix stiffening reported in lipedema.
Translational plausibility from related disease [28,32]	Clinical improvement of inflammatory and fibrotic endpoints in metabolic disease with tirzepatide.	MASH patients; HFpEF models	Provides human and translational signal that mechanisms relevant to lipedema are drug-responsive.
Proof-of-concept in lipedema (incretin class) [21]	Pilot study with GLP-1 receptor agonist (exenatide long-acting release—LAR) demonstrates metabolic and symptomatic benefits in women with lipedema and insulin resistance.	Small clinical series	Class signal supports moving to a more potent dual GIP/GLP-1 receptor co-agonist (tirzepatide) in lipedema trials.
Gynecologic interface [11,33]	Reviews highlight interest in incretins for hormone-dependent gynecologic disease due to anti-inflammatory and metabolic effects, including in polycystic ovary syndrome (PCOS) and endometriosis.	Narrative/umbrella reviews	Supports the lipedema–gynecology overlap (PCOS, endometriosis) as a context where tirzepatide may yield dual benefits.

**Table 2:** Chronological overview of the clinical and experimental development of tirzepatide, a dual co-agonist of the Glucose-Dependent Insulinotropic Polypeptide (GIP) and Glucagon-Like Peptide-1 (GLP-1) receptors, from early Phase III metabolic trials to recent translational studies in fibro-inflammatory diseases, illustrating the progressive evidence base supporting its potential relevance to lipedema. Abbreviations: GIP, Glucose-Dependent Insulinotropic Polypeptide; GLP-1, Glucagon-Like Peptide-1; T2D, Type 2 Diabetes; HbA1c, glycated hemoglobin; M1/M2, pro- and anti-inflammatory macrophage phenotypes; MASH, Metabolic Dysfunction–Associated Steatohepatitis; HFpEF, Heart Failure with Preserved Ejection Fraction.

Year	Study/Model	Population or Model	Key Outcomes	Relevance to Lipedema
2021	SURPASS-1(Rosenstock et al., The Lancet, 2021) [22]	Type 2 diabetes (T2D)	Significant reduction in glycated hemoglobin (HbA1c) by −2.3%, mean weight loss of 7.8–11.0 kg, and favorable safety profile.	Demonstrates strong metabolic and glycemic efficacy relevant to insulin-resistant lipedema phenotypes.
2022	SURMOUNT-1(Jastreboff et al., The New England Journal of Medicine, 2022) [23]	Obesity (non-diabetic participants)	Mean body weight reduction of 20.9% at 72 weeks; improved metabolic flexibility and energy expenditure.	Confirms unmatched weight loss and adipose tissue remodeling potential applicable to lipedema.
2024	Xia et al., International Immunopharmacology, 2024 [21]	Preclinical adipose macrophage model	Reduced M1 macrophage polarization, increased M2 anti-inflammatory phenotype, and improved insulin sensitivity.	Provides mechanistic support for anti-inflammatory action in lipedema-like adipose tissue.
2025	Hu et al., International Immunopharmacology, 2025 [27]	Metabolic dysfunction-associated steatohepatitis (MASH) model in diabetic mice	Reduced hepatic steatosis, inflammation, and fibrosis; modulated bile acid metabolism.	Demonstrates antifibrotic and immunometabolic effects translatable to lipedema pathophysiology.
2025	Packer et al., The New England Journal of Medicine, 2025 (HFpEF trial) [28]	Human participants with Heart Failure with Preserved Ejection Fraction (HFpEF) and obesity	Improved cardiac structure and function; reduced interstitial fibrosis; favorable tolerance and safety.	Confirms antifibrotic efficacy in a chronic fibro-inflammatory human condition relevant to lipedema.

## Data Availability

No new data were created or analyzed in this study. Data sharing is not applicable to this article.

## Abbreviations

17β-HSD	17β-Hydroxysteroid Dehydrogenase
ERα	Estrogen Receptor Alpha
ERβ	Estrogen Receptor Beta
GIP	Glucose-Dependent Insulinotropic Polypeptide
GLP-1	Glucagon-Like Peptide-1
HFpEF	Preserved Ejection Fraction
IL-6	Interleukin-6
M1 and M2	Macrophages
MASH	Metabolic-Associated Steatohepatitis
MCP-1	Monocyte Chemoattractant Protein-1
PCOS	Polycystic Ovary Syndrome
TNF-α	Tumor Necrosis Factor Alpha
UCP1	Uncoupling Protein 1
YKL-40	Chitinase-3-Like Protein 1
